# On Holobionts, Holospecies, and Holoniches: the Role of Microbial Symbioses in Ecology and Evolution

**DOI:** 10.1007/s00248-022-02005-9

**Published:** 2022-04-08

**Authors:** Roger T. Koide

**Affiliations:** Department of Biology, Brigham Young University, Provo, UT 84602 USA

**Keywords:** Adaptation, Conservation, Niche, Speciation, Species concept, Symbiosis

## Abstract

My goal in writing this is to increase awareness of the roles played by microbial symbionts in eukaryote ecology and evolution. Most eukaryotes host one or more species of symbiotic microorganisms, including prokaryotes and fungi. Many of these have profound impacts on the biology of their hosts. For example, microbial symbionts may expand the niches of their hosts, cause rapid adaptation of the host to the environment and re-adaptation to novel conditions via symbiont swapping, facilitate speciation, and fundamentally alter our concept of the species. In some cases, microbial symbionts and multicellular eukaryote hosts have a mutual dependency, which has obvious conservation implications. Hopefully, this contribution will stimulate a reevaluation of important ecological and evolutionary concepts including niche, adaptation, the species, speciation, and conservation of multicellular eukaryotes.

## Introduction

Many of the fundamental concepts in ecology and evolution assume symbiosis, the persistent association of two or more dissimilar species [1], is exceptional [2]. Yet symbioses involving microorganisms are the rule among multicellular eukaryotic organisms. Furthermore, microbial symbionts often have profound impacts on the biology of their eukaryote hosts. Indeed, the fundamental unit in the ecology of multicellular eukaryotes is most often not the eukaryote itself, but the holobiont [3, 4], collectively the eukaryote *and* its symbiotic microorganisms. Moreover, the holobiont is, in some cases, the unit on which natural selection acts [5]. No wonder Gilbert and colleagues [6] wrote “there have never been individuals.” Because symbioses with microorganisms so fundamentally influence the ecology and evolution of multicellular eukaryotes, I hope to provoke a reconsideration of several important concepts in light of microbial symbioses including the niche, adaptation, the species concept, speciation, and biological conservation.

## On Niches

Through the years, the term niche has been used to denote disparate concepts [7]. For the purposes of this contribution, therefore, I restrict my usage to the theoretical Hutchinson niche [8], defined as the n-dimensional niche space in which the species can maintain a population indefinitely and where the dimensions represent relevant environmental factors and resources. Hutchinson’s niche concept has proven to be useful in understanding various processes, including biological invasion [9], the maintenance of biological diversity [10], and the impact of climate change on communities [11].

The *fundamental niche*, assumed to be a unique property of each species [12], is conventionally viewed as being determined by a species’ genes as they interact with the environment. However, it has become obvious that a species’ niche is not necessarily the properties of the individual species. Consider a tree that can obtain sufficient phosphate from soils with some minimum phosphate availability, which defines one dimension or axis of the tree’s fundamental niche. When the tree associates with mycorrhizal fungi, however, it can obtain sufficient P from soils of significantly lower phosphate availability. The net result is that the tree can grow in many more habitats than it could in the absence of its mycorrhizal fungi.

In addition to expanding a niche along an existing resource axis, such as by lowering the required minimum soil phosphate availability just discussed, symbiosis can alter niches by producing entirely new traits in organisms. For example, the bean bug, *Riptortis pedestris,* can acquire insecticide resistance simply as a consequence of association with an insecticide-resistant intestinal bacterium [13]. There are many other examples of the acquisition of new traits due to symbiosis with microorganisms including nitrogen fixation in legumes [14], vitamin and amino acid production in sap-sucking insects [15], high-temperature tolerance in certain grasses [16], and cellulose digestion in ruminants [17] to list a few. Dawkins [18] referred to this phenomenon as one manifestation of the “extended phenotype.”

Historically, the *realized niche* was assumed to be a *subset* of the fundamental niche caused by negative interactions with other species that, for example, compete against the species of interest, thus reducing its niche volume [8]. However, we now appreciate that the symbiosis-mediated niche, arguably a type of realized niche, can actually be *larger* than the fundamental niche [19]. In referring to the ectomycorrhizal symbiosis, Peay [20] coined the term “mutualistic niche,” essentially an expanded fundamental niche*.* In an ecological sense, treating the mutualistic niche as an expanded fundamental niche rather than a kind of realized niche makes good sense. Plants and other multicellular eukaryotes do not exist in nature aposymbiotically, so there would appear to be no utility in referring to a species’ fundamental niche in the absence of its symbionts. In general, the symbiotically altered fundamental niche can be appreciated as the result of the hologenome [21], the collective genome of eukaryote and associated microorganisms. For clarity, this might be called the *holoniche* to be consistent with the term “holobiont.” The realized niche, then, is the result of the contraction of the holoniche due to, for example, the activity of competitors [8, 20].

Hutchinson [8] certainly did not consider symbioses as he formulated his niche concepts. However, because multicellular eukaryotes so commonly form symbioses with microorganisms that profoundly influence their niches, niche concepts now seem worthy of reconsideration. Moreover, the significant way in which microbial symbionts influence the niches of multicellular eukaryote species has major implications for adaptation, the species concept, speciation, and biological conservation.

## On Adaptation and Re-Adaptation via Symbiosis

As indicated above in the examples of acquired insecticide resistance by insects via gut bacteria [13], nitrogen fixation in legumes via root bacteria [14], amino acid production in insects via endosymbiotic bacteria [15], stress tolerance in plants via endophytic fungi [16], and cellulose digestion by ruminants via rumen microorganisms [17], eukaryotic organisms of all kinds acquire novel traits via symbioses with microorganisms that are not coded for by eukaryote genes. In an ecological context, these traits acquired through symbioses may be as relevant as intrinsic adaptations of the host alone. Moreover, compared to the evolution of intrinsic host adaptations, symbiotic trait acquisition may be far more rapid [22] and far more flexible in response to a fluctuating environment.

Indeed, symbiont modularity exists in some systems in which one symbiont can be lost and another gained (i.e., symbiont swapping), which may serve as a mechanism for rapid re-adaptation of the holobiont to novel conditions without evolution of the host per se. Consider the case of reef corals that associate with photosynthetic, dinoflagellate symbionts in the genus *Symbiodinium*. Environmental stresses, such as warming, may lead to “bleaching,” the loss of the photosynthetic symbionts, and, eventually, mortality of the coral and collapse of the ecosystem. However, the presence of stress-resistant *Symbiodinium* species in the water, even in low concentrations, may lead to the establishment of a novel holobiont with the ability to survive in warmer water, thus reestablishing ecosystem stability [23], despite a warming sea. Similarly, adaptive photo-symbiont switching occurs among fungi involved in lichen symbioses [24–26]. The desert woodrat (*Neotoma lepida*) currently maintains populations in the four major southwest American deserts (Mojave, Sonoran, Chihuahuan, and Great Basin deserts), but it encounters creosote bush (*Larrea tridentata*) in only the three southern-most deserts. It was once assumed that tolerance to toxic creosote bush resins by populations of the woodrat encountering the plant and the lack of tolerance to them by populations that do not encounter the plant were inherent traits of the woodrat, determined by its own genes [27]. More recently, it was shown that detoxication of the creosote bush resin is due to gut bacteria [28] and, remarkably, that gut bacteria transplantation can cause intolerant rats to suddenly tolerate creosote bush resins.

The ecological relevance of symbiotically acquired traits does not necessarily indicate that the holobiont is the unit of natural selection. Of course, when the eukaryote and its microbial symbionts have stable relationships, such as with sap-sucking insects and their strictly vertically transmitted endosymbiotic bacteria [29], it is easy to appreciate how the holobiont could evolve as a unit [6, 21], especially in the absence of conflicts of interest between symbionts and host [30]. However, in cases where the relationship between the eukaryote and its microbial symbionts is not stable through time, either because symbiont residence is highly transient for a given host individual or because the symbiont taxon is not stable across generations of the host, it would be difficult for the host and its microbial symbionts to evolve as a unit [31]. In symbioses with horizontally transmitted symbionts, particularly when the presence of multiple symbionts makes cheating possible [32], there is an increased likelihood that the holobiont is not the unit of natural selection. Nevertheless, this is not impossible [5].

Indeed, there are at least three reasons why some horizontally transmitted symbioses can achieve a relatively high degree of temporal stability. First, circumstances may substantially increase the likelihood that symbionts colonizing the parents will be horizontally transmitted to the offspring. For example, limited seed dispersal increases the likelihood that seedlings will develop near the parent plant and will, therefore, encounter the same endophytic [33] and mycorrhizal [34] fungi that colonize the parent. Second, while transmission of specific symbionts from parent to offspring is not guaranteed when it is horizontal, the establishment of specific symbionts may be incentivized when natural selection acts on the host to stabilize a particular association by rewarding the most beneficial symbionts [35] or sanctioning the less helpful [14]. Third, the transmission of specific symbionts from parent to offspring may not be necessary for selection to occur at the holobiont level when alternative symbiont species are functionally equivalent, and the host possesses little symbiont specificity. Such may be the case for at least a subset of the thousands of mycorrhizal fungal species capable of colonizing a single host plant species [36]. Therefore, it seems possible that, even when symbionts are horizontally transmitted, for some of these symbioses, the holobiont is the unit of natural selection.

Of course, the possibility of symbiosis-mediated adaptation does not preclude the evolution of intrinsic, adaptive host traits. Both are possible. The approach taken in any particular circumstance depends, of course, on which approach is most conveniently accomplished. In the film “Jurassic Park,” mathematician Ian Malcolm famously said, “life finds *a* way,” not “life finds *the* way.” Indeed, it may be possible for both intrinsic and symbiotic adaptations to develop simultaneously but, in cases where structural, developmental, or genetic constraints make the intrinsic adaptation impossible [37], symbiosis may save the day. Of course, if the symbiotic solution evolves first, it could obviate the evolution of the intrinsic adaptation.

## On Species

The notion that there are symbiotic solutions leading to the adaptation of eukaryotes to a variety of circumstances is clearly illustrated by the endosymbiotic bacteria of sap-sucking insects. Insects that have evolved to subsist on phloem sap obtain their nutrition from a source that is typically deficient in various essential amino acids [15]. One might expect natural selection to have remedied this untenable situation by fashioning metabolic pathways coded for by insect genes that manufacture the amino acids absent from their diet. Instead, these insects associate with particular bacterial lineages capable of producing the missing amino acids along with a range of other nutritive materials, including vitamins [15]. Some of these insect-bacterial associations have become tremendously intimate. In some of them, individual bacterial cells are packaged within individual host cells grouped together in an organ-like body, the bacteriome. But insect-bacteria symbioses can be even more involved; beta-proteobacterial symbionts may themselves host gamma-proteobacteria [38]. While the insects involved in these symbioses are nutritionally dependent on their bacteria, the bacteria have also become dependent on their host insects; in some cases, the genomes of the bacteria have become so reduced that necessary functions must be taken over by the host cells [15, 29]. Indeed, the bacterial cells are not able to survive outside of their hosts and must be transmitted vertically from insect to offspring. Vertically transmitted, endosymbiotic bacteria found in arbuscular mycorrhizal fungi also have reduced genome size and physiologic dependence on their fungal hosts [39–41]. These bacteria, in turn, may serve to increase fungal ATP production, stress tolerance, and reproductive output [39].

In such vertically transmitted symbioses, the bacteria and their eukaryote hosts are mutually dependent. Moreover, they have become so physically intimate that their association rivals that of mitochondria or chloroplasts within eukaryote cells [42–44]. None of us has any difficulty calling the soybean a unique species even if its chloroplasts were originally derived from an engulfed prokaryote. And yet we generally consider a sap-sucking insect and its bacterial symbionts to be separate species. When should we consider a symbiotic association a new, integrated species? Kiers and West [45] suggest that a new species exists when strict mutual dependence occurs among unique species, which is precisely the situation with so many of these intimate insect-bacteria symbioses. Therefore, in these cases, the insect and its bacteria comprise what could be termed a *holospecies*. In contrast, the legume-*Rhizobium* symbiosis has not yet turned this corner as it involves the horizontal acquisition of the bacteria by each generation of plants, allowing for there to be multiple bacterial genotypes in each root system and, therefore, the potential for cheating symbionts that confer no benefit [14].

## On Speciation

In an interview with Douglas Zook, Lynn Margulis indicated that symbiosis has produced new species by wholesale incorporation of entire novel genomes, what she referred to as symbiogenesis [46]. Symbiosis may also help produce new species via neo-Darwinian mechanisms. For example, speciation can be facilitated by genetic isolation of populations, which can be enforced by symbionts [47]. For example, genetically identical *Drosophila melanogaster* populations reared on different diets developed different intestinal microbial communities, which caused individuals from one population to discriminate against those from the other population in mating. The effect could be abolished with antibiotics and reestablished by inoculation [48]. Moreover, the structure of symbiotic bacterial communities in other animals is determined by gender, reproductive status, environment, genotype, and social relationships, so a knowledge of bacterial community structure can help in choosing mates [49–51]. Because the profile of bacterial secondary metabolites and their fermentation products functions as a signature of bacterial community structure, odors can be used to determine much about potential mates [49, 50].

Symbiosis can facilitate speciation via other neo-Darwinian mechanisms. When symbiosis causes an organism to occupy an expanded niche, it can also impact its geographic range. For example, fungal endophytes expand the geographic range of the grass *Bromus laevipes* into drier habitats [52]. When a symbiosis causes such a geographic range expansion [52, 53], it could facilitate reproductive isolation and, thus, contribute to speciation. Geographic range expansion can occur via other symbiosis-mediated mechanisms. Some gall midges associate with fungal symbionts. In fungal-symbiotic midges, eggs and fungal spores are simultaneously oviposited into plant tissues. Resultant galls are lined with fungal hyphae, which the midge larvae use as food. In contrast, larvae of non-symbiotic midges consume only plant tissues. In a comparison of fungal-symbiotic and non-symbiotic midge taxa, Joy [54] found that symbiotic lineages were significantly more diverse than their non-symbiotic relatives, possibly because of greater rates of speciation of symbiotic midges, which are not restricted to a small set of plant taxa they are able to consume directly as are the non-symbiotic midges.

## On Biological Conservation

Clearly, if eukaryotes are not anatomical, developmental, physiological, genetic, or immunological individuals but, rather, holobionts comprising the multicellular eukaryotes as well as their symbiotic microorganisms, the maintenance of biological diversity may be far more involved than what has been assumed. In addition to having goals regarding the conservation of eukaryote genome diversity, conserving the diversity of symbiotic microorganisms is also of obvious importance [55, 56], particularly for microbial species that are not vertically transmitted. Even when eukaryotes have vertically transmitted symbionts, there might still be conservation challenges for the symbiotic microorganisms. Because the population sizes of vertically transmitted microbial symbionts of animals or plants may be relatively small, mutations, including those resulting in gene loss or gene inactivation leading to functional incompetence, could readily become fixed in the microbial population.

The suitability of potential foods for many animals from woodrats to koalas to colobus monkeys is determined, in part, by symbiotic gut microorganisms [28, 57, 58]. For horizontally transmitted microbial symbionts, such as much of the intestinal microbiota, appropriate microbial symbionts must be acquired from the environment. Consider the conservation of animal species in captivity or plant species in seed banks. Because artificial environments may strongly influence the gut microbiota of vertebrate animals [55, 56], captive-reared animals may not be microbiologically equipped to function as required in the wild. The same problem may occur when we attempt to transfer animals from one habitat to another [59]. Moreover, altering an established but inappropriate gut microbial community to that of an appropriate community may not be easy because of priority effects [60], even if some of the appropriate microorganisms are available from a wild food source [61]. And, because seeds germinated in locations where plants of the same species have been absent for long periods may lack symbiotic microorganisms specific to that species [62], species conserved in seed banks may be similarly ill-equipped to survive in natural ecosystems. It is possible that appropriate microbial communities would develop with sufficient time. The question, of course, is whether they would develop rapidly enough to maintain a sustainable population of hosts in the wild.

Communication among individual animals that ensure reproductive success, such as that which indicates species, social group, and gender, is sometimes mediated by volatile compounds produced by microbial symbionts [63]. Thus, inappropriate symbiotic microbial communities developing as a consequence of rearing in artificial environments [55] could also reduce the likelihood of sustaining viable eukaryote populations in the wild.

## Concluding Remarks

Although documentation of particular examples of symbiosis dates from the late nineteenth century [64–66], symbioses continue to be treated largely as if they were exceptional [2]. Consequently, the basis for many of our cherished ecological and evolutionary concepts, including the niche, adaptation, the species concept, speciation, and conservation, was developed largely without an appreciation of the many important roles played by symbiotic microorganisms. It is clear, however, that symbioses are not exceptional; virtually, all multicellular eukaryote species form intimate symbioses with microorganisms that strongly influence the ecology and evolution of the multicellular eukaryote. Therefore, the hope is that this contribution will serve to promote research in microbial ecology with a fuller appreciation for the vital role that microorganisms play in eukaryote ecology and evolution.

## Data Availability

Not applicable.

## References

[1] Oliver KM, Russell JA (2016). Symbiosis, Introduction to. Encycl Evol Biol.

[2] Sapp J (2004). The dynamics of symbiosis: an historical overview. Can J Bot.

[3] Bordenstein SR, Theis KR (2015). Host biology in light of the microbiome: ten principles of holobionts and hologenomes. PLoS Biol.

[4] Dittami SM, Arboleda E, Auguet JC (2021). A community perspective on the concept of marine holobionts: current status, challenges, and future directions. PeerJ.

[5] Roughgarden J (2020). Holobiont evolution: mathematical model with vertical vs. horizontal microbiome transmission. Philos Theory Pract Biol.

[6] Gilbert SF, Sapp J, Tauber AI (2012). A symbiotic view of life: we have never been individuals. Q Rev Biol.

[7] Whittaker R, Levin S, Root R (1973). Niche, habitat, and ecotope. Am Nat.

[8] Hutchinson G (1957). Concluding remarks. Cold Spring Harb Symp Quant Biol.

[9] Broennimann O, Treier UA, Müller-Schärer H (2007). Evidence of climatic niche shift during biological invasion. Ecol Lett.

[10] Grubb P (1977). The maintenance of species-richness in plant communities: the importance of the regeneration niche. Biol Rev.

[11] Cang FA, Wilson AA, Wiens JJ (2016). Climate change is projected to outpace rates of niche change in grasses. Biol Lett.

[12] Colwell RK, Rangel TF (2009). Hutchinson’s duality: the once and future niche. Proc Natl Acad Sci USA.

[13] Kikuchi Y, Hayatsu M, Hosokawa T (2012). Symbiont-mediated insecticide resistance. Proc Natl Acad Sci.

[14] Kiers ET, Rousseau RA, West SA, Denison RF (2003). Host sanctions and the legume-rhizobium mutualism. Nature.

[15] Moran NA, Bennett GM (2014). The tiniest tiny genomes. Annu Rev Microbiol.

[16] Rodriguez RJ, Redman RS, Henson JM (2004). The role of fungal symbioses in the adaptation of plants to high stress environments. Mitig Adapt Strateg Glob Chang.

[17] Russell JB, Muck RE, Weimer PJ (2009). Quantitative analysis of cellulose degradation and growth of cellulolytic bacteria in the rumen. FEMS Microbiol Ecol.

[18] Dawkins R (1999). The extended phenotype: the long reach of the gene.

[19] Bulleri F, Bruno JF, Silliman BR, Stachowicz JJ (2016). Facilitation and the niche: Implications for coexistence, range shifts and ecosystem functioning. Funct Ecol.

[20] Peay KG (2016). The mutualistic niche: mycorrhizal symbiosis and community dynamics. Annu Rev Ecol Syst.

[21] Rosenberg E, Zilber-Rosenberg I (2018). The hologenome concept of evolution after 10 years. Microbiome.

[22] Alberdi A, Aizpurua O, Bohmann K (2016). Do vertebrate gut metagenomes confer rapid ecological adaptation?. Trends Ecol Evol.

[23] Baker AC (2003). Flexibility and specificity in coral-algal symbiosis: diversity, ecology, and biogeography of Symbiodinium. Annu Rev Ecol Evol Syst.

[24] Piercey-Normore MD, DePriest PT (2001). Algal switching among lichen symbioses. Am J Bot.

[25] Ertz D, Guzow-Krzemińska B, Thor G (2018). Photobiont switching causes changes in the reproduction strategy and phenotypic dimorphism in the Arthoniomycetes. Sci Rep.

[26] Lücking R, Lawrey JD, Sikaroodi M (2009). Do lichens domesticate photobionts like farmers domesticate crops? Evidence from a previously unrecognized lineage of filamentous cyanobacteria. Am J Bot.

[27] Mangione A, Dearing M, Karasov W (2000). Interpopulation differences in tolerance to creosote bush resin in desert woodrats (Neotoma lepida). Ecology.

[28] Kohl KD, Weiss RB, Cox J (2014). Gut microbes of mammalian herbivores facilitate intake of plant toxins. Ecol Lett.

[29] McCutcheon JP, Von Dohlen CD (2011). An interdependent metabolic patchwork in the nested symbiosis of mealybugs. Curr Biol.

[30] Figueiredo ART, Kramer J (2020). Cooperation and conflict within the microbiota and their effects on animal hosts. Front Ecol Evol.

[31] Stévenne C, Micha M, Plumier JC, Roberty S (2021) Corals and sponges under the light of the holobiont concept: how microbiomes underpin our understanding of marine ecosystems. Front Mar Sci 8. 10.3389/fmars.2021.698853

[32] Lekberg Y, Koide RT (2014). Integrating physiological, community, and evolutionary perspectives on the arbuscular mycorrhizal symbiosis. Botany.

[33] Ricks KDKD, Koide RTRT (2019). The role of inoculum dispersal and plant species identity in the assembly of leaf endophytic fungal communities. PLoS ONE.

[34] Liang M, Johnson D, Burslem DFRP (2020). Soil fungal networks maintain local dominance of ectomycorrhizal trees. Nat Commun.

[35] Kiers ET, Duhamel M, Beesetty Y (2011). Reciprocal rewards stabilize cooperation in the mycorrhizal symbiosis. Science.

[36] Trappe J (1977). Selection of fungi for ectomcorrhizal inoculation in nurseries. Annu Rev Phytopathol.

[37] Gould S, Lewontin R (1979). The spandrels of San Marco and the Panglossian paradigm: a critique of the adaptationist programme. Proc R Soc B Biol Sci.

[38] von Dohlen C, Kohler S, Alsop ST, McManus WR (2001). Mealybug β-proteobacterial endosymbionts contain γ-proteobacterial symbionts. Nature.

[39] Salvioli A, Ghignone S, Novero M, et al (2015) Symbiosis with an endobacterium increases the fitness of a mycorrhizal fungus, raising its bioenergetic potential. ISME J 1–15. 10.1038/ismej.2015.91 10.1038/ismej.2015.91 PMC4681866 26046255

[40] Torres-Cortés G, Ghignone S, Bonfante P, Schüßler A (2015) Mosaic genome of endobacteria in arbuscular mycorrhizal fungi: transkingdom gene transfer in an ancient mycoplasma-fungus association. Proc Natl AcadSci 201501540. 10.1073/pnas.1501540112 10.1073/pnas.1501540112 PMC4485150 25964335

[41] Naito M, Morton JB, Pawlowska TE (2015) Minimal genomes of mycoplasma-related endobacteriaare plastic and contain host-derived genes for sustained life within Glomeromycota. Proc Natl Acad Sci 201501676. 10.1073/pnas.1501676112 10.1073/pnas.1501676112 PMC4485128 25964324

[42] Taylor FJR (1987). An overview of the status of evolutionary cell symbiosis theories. Ann N Y Acad Sci.

[43] Kuo C-H (2015). Scrambled and not-so-tiny genomes of fungal endosymbionts. Proc Natl Acad Sci.

[44] Keeling PJ, McCutcheon JP, Doolittle WF (2015). Symbiosis becoming permanent: Survival of the luckiest. Proc Natl Acad Sci USA.

[45] Kiers BET, West SA (2015). Evolving new organisms via symbiosis. Science.

[46] Zook D (2015) Symbiosis - evolution’s co-author. In: Gontier N (ed) Reticulate evolution, symbiogenesis, lateral gene transfer, hybridization and infections heredity, interdisciplinary evolution research 3. Springer, Cham, pp 41–80

[47] Brucker RM, Bordenstein SR (2012). Speciation by symbiosis. Trends Ecol Evol.

[48] Sharon G, Segal D, Ringo JM (2010). Commensal bacteria play a role in mating preference of Drosophila melanogaster. Proc Natl Acad Sci.

[49] Shropshire JD, Bordenstein SR (2016). Speciation by symbiosis: the microbiome and behavior. MBio.

[50] Archie EA, Theis KR (2011). Animal behaviour meets microbial ecology. Anim Behav.

[51] Leclaire S, Nielsen JF, Drea CM (2014). Bacterial communities in meerkat anal scent secretions vary with host sex, age, and group membership. Behav Ecol.

[52] Afkhami ME, Mcintyre PJ, Strauss SY (2014). Mutualist-mediated effects on species’ range limits across large geographic scales. Ecol Lett.

[53] Kazenel MR, Debban CL, Ranelli L (2015). A mutualistic endophyte alters the niche dimensions of its host plant. AoB Plants.

[54] Joy JB (2013). Symbiosis catalyses niche expansion and diversification. Proc R Soc B Biol Sci.

[55] Carthey AJR, Blumstein DT, Gallagher R V., et al (2019) Conserving the holobiont. Funct Ecol 764–776. 10.1111/1365-2435.13504

[56] Trevelline BK, Fontaine SS, Hartup BK, Kohl KD (2019) Conservation biology needs a microbial renaissance: a call for the consideration of host-associated microbiota in wildlife management practices. Proc R Soc B Biol Sci 286. 10.1098/rspb.2018.2448 10.1098/rspb.2018.2448 PMC6364583 30963956

[57] Blyton MDJ, Soo RM, Whisson D (2019). Faecal inoculations alter the gastrointestinal microbiome and allow dietary expansion in a wild specialist herbivore, the koala. Anim Microbiome.

[58] Barelli C, Albanese D, Donati C (2015). Habitat fragmentation is associated to gut microbiota diversity of an endangered primate: implications for conservation. Sci Rep.

[59] Kohl KD, Varner J, Wilkening JL, Dearing MD (2018). Gut microbial communities of American pikas (Ochotona princeps): evidence for phylosymbiosis and adaptations to novel diets. J Anim Ecol.

[60] Nettles R, Ricks KD, Koide T, Koide RT (2020). The dynamics of interacting bacterial and fungal communities of the mouse colon following antibiotics. Microb Ecol.

[61] Kohl KD, Dearing MD (2014). Wild-caught rodents retain a majority of their natural gut microbiota upon entrance into captivity. Environ Microbiol Rep.

[62] Ricks KD, Koide RT (2019). Biotic filtering of endophytic fungal communities in Bromus tectorum. Oecologia.

[63] Ezenwa VO, Williams AE (2014). Microbes and animal olfactory communication: Where do we go from here?. BioEssays.

[64] de Bary A (1879). Die Erscheinung der Symbiose: Vortrag, Gehalten auf der Versammlung Deutscher Naturforscher und Aerzte zu Cassel.

[65] Frank B (2005). On the nutritional dependence of certain trees on root symbiosis with belowground fungi (an English translation of A.B. F rank’s classic paper of 1885). Mycorrhiza.

[66] Berch SM, Massicotte HB, Tackaberry LE (2005). Re-publication of a translation of “The vegetative organs of Monotropa hypopitys L”. published by F. Kamienski in 1882, with an update on Monotropa mycorrhizas. Mycorrhiza.

